# High-speed force spectroscopy: microsecond force measurements using ultrashort cantilevers

**DOI:** 10.1007/s12551-019-00585-4

**Published:** 2019-10-07

**Authors:** Claire Valotteau, Fidan Sumbul, Felix Rico

**Affiliations:** Aix-Marseille Univ, INSERM, CNRS, LAI, 13009 Marseille, France

**Keywords:** Atomic force microscopy, High-speed atomic force microscopy, HS-AFM, High-speed force spectroscopy, HS-FS, Ultrashort cantilevers, Molecular mechanics, Cellular mechanics

## Abstract

Complete understanding of the role of mechanical forces in biological processes requires knowledge of the mechanical properties of individual proteins and living cells. Moreover, the dynamic response of biological systems at the nano- and microscales span over several orders of magnitude in time, from sub-microseconds to several minutes. Thus, access to force measurements over a wide range of length and time scales is required. High-speed atomic force microscopy (HS-AFM) using ultrashort cantilevers has emerged as a tool to study the dynamics of biomolecules and cells at video rates. The adaptation of HS-AFM to perform high-speed force spectroscopy (HS-FS) allows probing protein unfolding and receptor/ligand unbinding up to the velocity of molecular dynamics (MD) simulations with sub-microsecond time resolution. Moreover, application of HS-FS on living cells allows probing the viscoelastic response at short time scales providing deep understanding of cytoskeleton dynamics. In this mini-review, we assess the principles and recent developments and applications of HS-FS using ultrashort cantilevers to probe molecular and cellular mechanics.

## Introduction

Mechanical forces play an essential role in many biological processes at different length scales, from nanometers in force bearing adhesion molecular complexes to micrometers in contractile muscle cells (Boal and Boal 2012). This wide range of length scales combines with a wide range of timescales, from sub-millisecond conformational changes in individual biomolecules to minutes or even hours-long processes in living cells. Thus, full understanding of the mechanical properties of biological systems requires a tool capable of accessing these wide ranges of length and time scales. Atomic force microscopy (AFM) is now well established for the visualization of biological systems in action at near physiological conditions and with nanometer resolution (Krieg et al. 2019). Force spectroscopy (FS) emerged early after AFM invention and was quickly applied to biological systems. In this mode, the AFM cantilever is used as a force sensor to probe the mechanics of individual biomolecules and living cells with piconewton force resolution (Rief et al. 1997; Florin et al. 1994; Moy et al. 1994; Fritz et al. 1998; Hinterdorfer et al. 1996; Radmacher et al. 1992; Hoh et al. 1992). While AFM is a widely used technique, the time resolution of force measurements is limited by the response time of the AFM components, mainly the cantilever, commonly in the millisecond (ms) range. However, many biological processes, such as molecular binding, transmembrane channel gating or protein (un)folding, especially at the single molecule level, occur at shorter times, down to the micro- and nanosecond timescales. Recent work using optical tweezers allowed the observation of fast biomolecular processes with a resolution of ~ 10 μs (Neupane et al. 2016). The development of high-speed AFM (HS-AFM) using cantilevers with μs-response time allowed an increase in imaging rates by about 1000-fold, providing a new tool to visualize protein and cellular dynamics at video rates (Ando et al. 2001; Humphris et al. 2005; Viani et al. 1999). The developments by the groups of Hansma and Ando were based on the miniaturization of the main AFM components, such as piezoelectric elements and, especially, adapted optics allowing the use of ultrashort cantilevers (Ando et al. 2001, 2008; Walters et al. 1997, 1996; Schaeffer et al. 1997). HS-AFM allowed, for example, to the landmark observation of myosin V walking on an actin filament in real time (Kodera et al. 2010). Other dynamic processes reported using HS-AFM include the glass-like diffusion of membrane proteins, the formation of protein complexes on membranes and the light-induced conformational changes of proton pumps (Ando et al. 2008; Casuso et al. 2012; Colom et al. 2013; Shibata et al. 2010; Chiaruttini et al. 2015; Munguira et al. 2016; Viani et al. 2000). HS-AFM is thus an excellent tool to study biological molecules in action thanks to its high spatiotemporal resolution (Ando 2018; Ando et al. 2014). The application of ultrashort cantilevers for force spectroscopy was also considered from the very beginning as they provided enhanced force precision and μs time resolution (Viani et al. 1999; Gutsmann et al. 2004). Thus, high-speed force spectroscopy (HS-FS) with μs time resolution emerged as a unique tool to observe rare and short events during biomolecular processes, such as intermediate states during protein unfolding or bond rupture. Moreover, it provided at long-awaited method to reach the velocities and timescales of all atom molecular dynamics (MD) simulations which are orders of magnitude away from experimental ones but essential to understand the atomic details of biomolecular processes (Izrailev et al. 1997; Sotomayor and Schulten 2007). In this short review, we discuss the most recent developments and applications in high-speed force spectroscopy (HS-FS), which relies on the use of ultrashort HS-AFM cantilevers for force measurements with microsecond time resolution.

## Ultrashort cantilevers and HS-AFM

Ultrashort cantilevers provide advantages that make them optimal for force measurements with high temporal and force resolution: moderate spring constant (k), low quality factor (Q), reduced viscous drag coefficient (β), and short response time (τ). As shown below, these parameters are interdependent. For a cantilever of rectangular shape, the spring constant is determined by the material properties (Young’s modulus, E) and dimensions (length *L*, width *w* and thickness *t*) by $k=\frac{Ew{t}^3}{4{L}^3}$ (notice the cubic dependence of length and thickness). Thus, short lengths imply high spring constants and, to achieve moderate *k*, thin cantilevers are required. The dynamic response of a cantilever is often described in terms of the simple harmonic oscillator of effective mass (*m*) and spring constant (*k*), whose resonance frequency *f*_0_ of the first oscillation mode is given by

1 $$ {\omega}_0=2\pi {f}_0=\sqrt{\frac{k}{m}} $$

It is important to notice that *m* is an effective mass, which does not coincide with the mass of the cantilever and is affected by the viscosity and density of surrounding medium (Morse 1983; Timoshenko 1937; Leissa 1969; Sader 1998). This effective mass is proportional to the volume of the cantilever, thus, *ω*_0_ ∝ *t*/*L*^2^ (also dependent on the medium properties). Therefore, light mass and a relatively stiff spring constant is required for high resonance frequencies and, consequently, short response times. The quality factor *Q*, or the number of oscillations before complete damping out, is proportional to the spring constant and inversely proportional to the viscous drag coefficient and the resonance frequency:

2 $$ Q=\frac{k}{\omega_0\beta } $$

or, using Eq. 1,

3 $$ Q=\frac{\omega_0m}{\beta } $$

The quality factor importantly determines the response time of the cantilever

4 $$ \tau =\frac{Q}{\pi {f}_0} $$

**Fig. 1:**
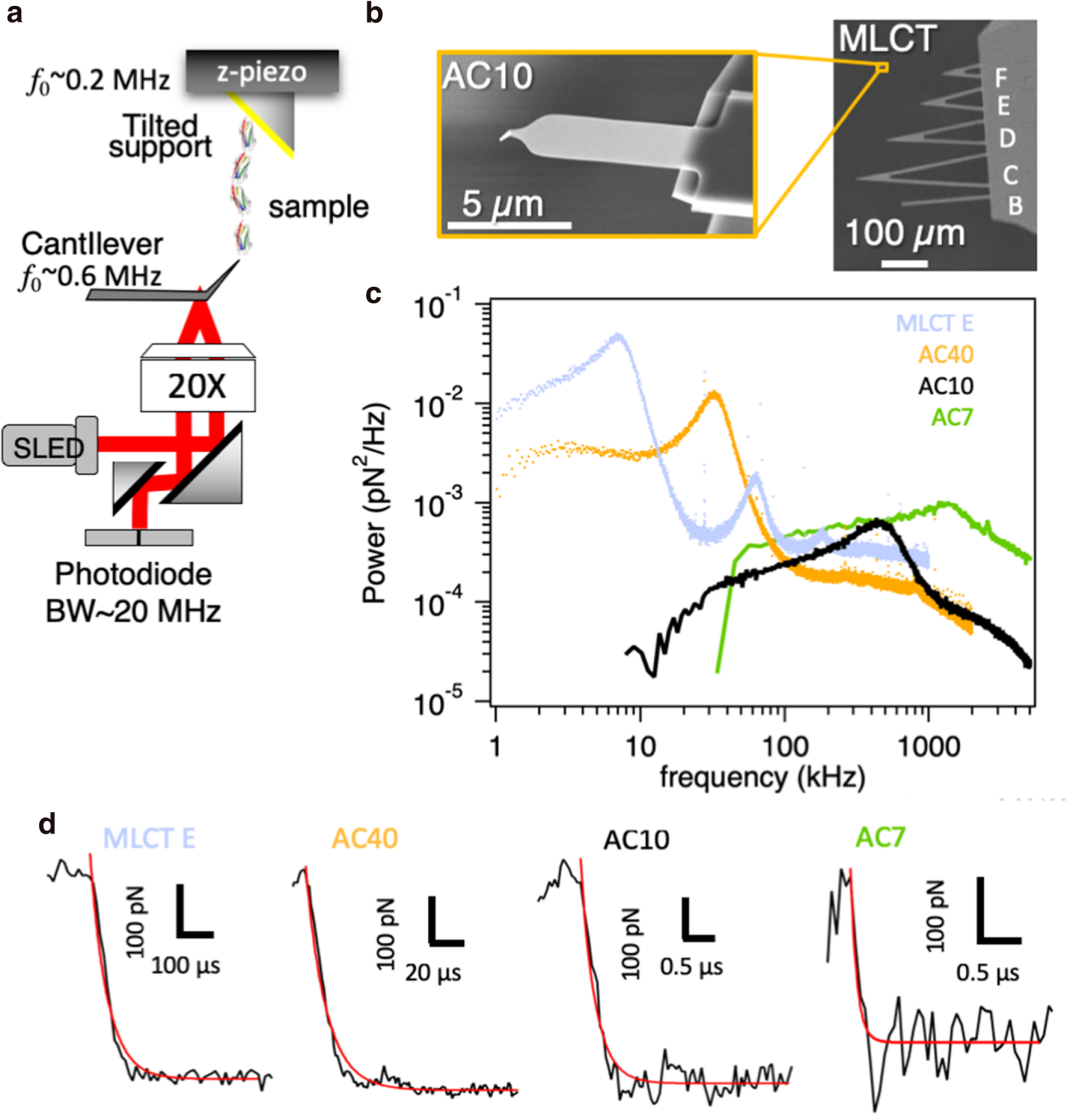
High-speed force spectroscopy. **a** Example of HS-AFM setup for force spectroscopy. The resonance frequency or the bandwidth (BW) of the piezoelectric element, cantilever and photodiode are shown. **b** Electron micrograph of ultrashort (AC10) compared with conventional cantilevers (MLCT), revealing the reduced dimensions. **c** Thermal spectra after removal of 1/f noise and **d** time response of conventional (MLCT-E and AC40) and ultrashort cantilevers (AC10 and AC7). Cantilever parameters are listed in Table 1

Notice that low quality factors and high resonance frequencies are required to achieve short response times. The viscous drag coefficient determines, not only the response time, but, consequently, the maximum velocity that the cantilever can reach. The viscous drag coefficient depends on the geometry and dimensions of the cantilever, mainly on its plan area. Thus, short cantilevers provide low *β*, featuring high *k*. For example, shortening the length of a rectangular cantilever by two will lead to ~ 2 times lower viscous drag coefficient but ~ 8 times higher spring constant. Importantly, the viscous drag coefficient is inversely proportional to the separation between the cantilever and the substrate (Alcaraz et al. 2002; Janovjak et al. 2005). Thus, accurate determination of the viscous drag coefficient requires measurement of the viscous drag forces at different distances from the substrate. An elegant approach is to oscillate the cantilever at low amplitude while approaching the tip to the substrate, as it provides an almost continuous value of *β* as a function of the separation distance (Sunyer et al. 2009). Apart from low viscous drag coefficients, ultrashort cantilevers in liquid present low quality factors (*Q*~1). Accurate experimental determination of the quality factor is difficult when the quality factor itself is low. Moreover, near the substrate, cantilevers present lower resonance frequency and lower Q than in the bulk (Benmouna and Johannsmann 2002). This is also the case for ultrashort cantilevers (Rigato et al. 2017). Thus, both *Q* and *β* depend on the topography of the sample and their accurate determination is to be done at the exact point of force measurement. This is important to accurately determine the response time. Thus, while the response time of a cantilever may be derived from the equations above using *f*_0_ and *Q* from the thermal spectrum in bulk, the actual response time at the point of measurement will likely vary from this value, especially when the cantilever is near the surface. An alternative approach to determine the response time involves fitting an exponential decay to the force-time response after a force step at the specific tip-sample separation as shown in Fig. 1.

The force resolution (ΔF) is also an essential parameter at short timescales, as it determines the minimum force that can be effectively measured by the cantilever. The force precision of a cantilever at the micrometer scale depends, not on its spring constant, but on its viscous drag coefficient, on the bandwidth of the measurement (Δf) and on the thermal energy (*k*_B_*T*, with *k*_B_ the Boltzmann’s constant and *T* the absolute temperature)

5 $$ \Delta F=\sqrt{4{k}_BT\Delta f\beta} $$

While the force resolution is improved using ultrashort cantilevers, it depends on the bandwidth, *i.e*. the timescale, of the measurement. To measure forces at short timescales in the μs regime, wide bandwidths in the MHz regime are required, which lowers the force resolution. Given Eqs. 4 and 5, to achieve even shorter response times and improved force precision, it seems reasonable to reduce the *Q* factor and/or the viscous drag coefficient. This strategy led to the application of ultrashort cantilevers for high-speed force spectroscopy, as described below.

It is important to note that, in addition of the thermal noise described by Eq. 5, the deflection sensitivity of the optical lever system, the spring constant of the cantilever and electronic noise due to the photodiode and associated electronics indirectly affect the force precision. Indeed, for cantilevers with high spring constant, the signal to noise ratio is poor compared with softer levers, due to the lower signal (lower deflection) at a same level of force. This is reflected, for example, in the higher background noise of AC7 cantilevers compared with AC10, where same electronics were used (Fig. 1). Thus, minimized electronic noise, high deflection sensitivity, and cantilevers with moderate spring constants also improve force resolution.

Current ultrashort cantilevers have typical dimensions of ~ 8 μm long by ~ 2 μm wide by 0.1 μm thick, about 10 times smaller than conventional AFM cantilevers. The thermal spectra and the time response of various types of cantilevers commonly used in force spectroscopy are shown in Fig. 1. The relevant cantilever parameters described above are reported in Table 1 for the same cantilevers. The advantage of ultrashort cantilevers (AC10 and AC7 models) is clear, as they feature resonance frequencies of ~ 500 kHz and ~ 1200 kHz, Q-factors ~ 1, and response times ~ 0.2 μs and ~ 0.1 μs, respectively, in liquid. Moreover, they are the mostly used probes for HS-FS due to the material of the tip (silicon nitride) which allows better functionalization as compared with the carbon of electron beam deposited tips.

**Table 1:** Properties of commonly used conventional and ultrashort cantilevers

		Cantilever model			
		MLCT-E	AC40	AC10	AC7
Shape		V	rect	rect	rect
Length (μm)		140	38	8	6
Width (μm)		18	16	2	2
Thickness (nm)		600	180	130	130
*k*^1^ (pN/nm)		112	102	87	592
*f*_0_ in liquid (kHz)^2^		7	31	431	1231
Q-factor in liquid^2^		1.7	1.6	0.8	0.7
*β* (pN·s/μm)	Eq. 4	4.59	0.82	0.03	0.05
*τ* (μs)	Eq. 4	82	16	0.62	0.18
	Experiment	44	17	0.22	0.07

^1^*k* was determined from the Sader method in air (Sader et al. 2012)

^2^*f*_*0*_ and *Q* were determined from the Lorenztian fit to the thermal spectrum

Apart from ultrashort cantilevers, HS-AFM and HS-FS require dedicated instrumentation (Ando et al. 2008). Advanced electronics for fast force feedback is necessary for HS-AFM video rate imaging at a precisely controlled applied force (Kodera et al. 2006). In addition, high-frequency piezoelectric elements (with resonance frequencies of between 30 and 200 kHz) allow the application of high velocities (up to mm/s) although within limited travel distances (< 1 μm). Finally, the reduced dimensions of ultrashort cantilevers require an optical lever detection system with a small laser spot. Indeed, the main development of AFM systems to allow the use of ultrashort cantilevers was the modification of the optical lever detection system. High power microlenses or microscope objectives are currently used to allow focusing the laser beam onto a spot of diameter of 2–3 μm, comparable with the width of the cantilever (Ando et al. 2001, 2008; Schäffer et al. 1996; Fantner et al. 2006). Despite their advantages, due to the requirement of specialized optics, the use of ultrashort cantilevers is limited to a reduced number of commercial AFM systems (RIBM Ando-type, Cypher VRS and FastScan Bruker).

## Developments for high-speed force spectroscopy

To better adapt HS-AFM systems and ultrashort cantilevers for HS-FS measurements, different developments have been implemented. As described above, the temporal and force resolution of HS-FS measurements depends on the geometry of the cantilevers. Commercially available ultrashort HS-AFM cantilevers (Table 1) are not optimal for force measurements. While they provide excellent time resolution (sub-μs), ultrashort cantilevers may present limited force precision performance and “ringing” in the force response (Edwards et al. 2017). In addition, close proximity to the substrate leads to an important increase in the effective viscous drag, which may limit fast loading velocities. Improved force precision by lowering the viscous drag coefficient will facilitate detection of small and fast molecular changes and rare events in force spectroscopy measurements. A simple approach to achieve that is reducing the effective separation between the cantilever and the substrate using tilted sample supports (Fig. 1a). This strategy was used in our first application of ultrashort cantilevers for protein unfolding (Rico et al. 2013), reducing the viscous drag coefficient by a factor of two, and allowing the application of high velocities, in the mm/s regime.

Another method of improving the performance of AFM cantilevers is the use of focused ion-beam (FIB) technology to modify the geometry and dimensions of the cantilever (Hodges et al. 2001; Bull et al. 2014). FIB milling has been employed to improve the force stability and precision while maintaining an excellent temporal resolution. The group of Perkins has modified and tested different milling strategies on commercially available cantilevers to decrease the stiffness and the hydrodynamic drag coefficient (Edwards et al. 2017, 2015; Bull et al. 2014; Edwards and Perkins 2017). Indeed, an important assumption in single molecule force spectroscopy (SMFS) analysis methods is the use of overdamped force probes (*Q* < 0.5) (Bell 1978; Dudko et al. 2006; Evans and Ritchie 1997; Merkel et al. 1999). Ultrashort HS-AFM cantilevers feature quality factors in liquid ~ 1, which violates the underlying assumption of an overdamped force probe (Edwards et al. 2015; Sumbul et al. 2018a). Underdamped probes may oscillate at their resonance frequency causing “ringing” effects after a sharp force step. This oscillation may prevent the detection of short-lived, minute changes in the biomolecule structure during single molecule experiments (Faulk et al. 2017). Via FIB milling, the *Q* factor of ultrashort high-speed AFM cantilevers was reduced to *Q* < 0.5, assuring overdamped response, while improving force stability and maintaining 1-μs-scale temporal resolution.

Another aspect that affects the force stability is the drift in the force arising from the cantilever’s gold coating due to the bimetallic structure. Minimal gold coating reduces the force drift during the measurements. Bull et al. successfully minimized drift of commercial cantilevers by preserving a small area of gold coating at the very end of the cantilever covering it with tetraethyl orthosilicate (TEOS). This allowed keeping a reflective surface while reducing the adverse effect of the bimetallic stress and achieving sub-pN force precision over five decades of bandwidth (0.01 Hz–1000 Hz) (Bull et al. 2014). The application of this method to ultrashort cantilevers would also improve the force precision and stability at the shortest timescales.

Apart from modification of the HS-AFM system and cantilevers, the preparation of the sample is an essential bottleneck in FS experiments. In commercially available HS-AFM setups and cantilevers, the type and the size of the sample support as well as the dimensions of the probe are limited. For example, ultrashort cantilevers commonly feature sharp tips, good for imaging, but which lead to low success rate in single molecule HS-FS measurements. Thus, more robust techniques for protein immobilization are important to improve binding efficiency and reproducibility. Moreover, the commonly used gold substrates for thiol grafting of proteins lead to important interference artifacts (Edwards et al. 2015). The recent discovery of mechanically ultra-stable receptor/ligand bonds, like those formed in dockerin/cohesin III (Schoeler et al. 2014) and Fgß/SdrG (Milles et al. 2018) complexes, now allows protein unfolding experiments by grabbing the molecules from specific sites, with precise knowledge of the pulling direction and with higher efficiency than previous methods based on unspecific attachment. Importantly, these strategies are compatible with low reflective surfaces such as glass or mica, which minimize optical interference artifacts. Therefore, using ultra-stable molecular complexes turns out to be an excellent approach for HS-FS measurements (Ott et al. 2016). Finally, working on living cells requires combining the HS-AFM with advanced optical microscopy techniques and modified cantilever tips. Modification of current setups or coupling the HS-AFM systems with inverted optical microscopes has been already carried out in the recent years, allowing precise positioning of the probe on the cell surface and combination with molecular information using fluorescence markers (Colom et al. 2013; Suzuki et al. 2013; Fukuda et al. 2013; Shibata et al. 2015; Yamashita et al. 2012).

## Possible or required developments for improved force and time resolution

While remarkable achievements have been accomplished thanks to use of ultrashort cantilevers and fast piezoelectric elements, some improvements are still necessary. Calibration of the AFM cantilevers spring constant and of the AFM setup optical lever sensitivity (OLS) is one of the most important steps in force spectroscopy measurements. The available techniques used to calibrate AFM cantilevers can be classified in two: contact and noncontact methods. Contact based methods require acquiring force-distance curves on a hard surface to determine the OLS. In order to get a reliable value, the force applied should be high enough to minimize interfacial surface forces. Therefore, there is a risk to damage the cantilever tip and more importantly its coating. After determination of the OLS, the thermal method is commonly used to determine k (Hutter and Bechhoefer 1993; Butt and Jaschke 1995). Conversely, noncontact methods require prior knowledge of spring constant of the cantilever and use the thermal spectrum for OLS determination. Thus, noncontact methods prevent tip damage and have been shown to be more accurate, but require pre-calibrated cantilevers (Schillers et al. 2017; Higgins et al. 2006). There are different methods to determine the spring constant of a cantilever. Some of them, like the use of a vibrometer, may be difficult to implement in practice and require expensive instrumentation (Schillers et al. 2017). Another method was developed by Sader et al. (1999, 1995) and is easily implemented in almost any AFM system. However, it is valid only for high Q-factor cantilevers, *i.e*., for ultrashort cantilevers, in air, but not in liquid. Recently, Sader and coworkers initiated a web-based platform for spring constant calibration, the global calibration initiative (GCI) (Sader et al. 2016) in which users are able to upload their own calibrations to the database and/or get the calculated spring constant from the website by only uploading the resonance frequency and Q-factor of the cantilever. The accuracy of this method is expected to increase while the pool of calibrated cantilever data enlarges and may enable a dynamic process of recalibration.

As mentioned before, due to the movement of the surrounding fluid relative to the cantilever, a viscous drag force is generated on the cantilever during force spectroscopy measurements. This force is proportional to the viscous drag coefficient and the velocity of the cantilever and causes a deviation in applied force. Therefore, especially at very high velocities, this viscous drag effect should be corrected (Janovjak et al. 2005; Rico et al. 2019). Moreover, recent theoretical predictions suggest that the viscous drag of the cantilever may affect the kinetics and measured forces (rupture/unfolding) on single molecule force spectroscopy measurements (Cossio et al. 2016). Thus, cantilevers with even lower viscous drag coefficients may be necessary to further improve our understanding of the mechanics of single molecules. The use of milled cantilevers or tilted supports, as exposed previously, is a first step in this direction. Substrate tilting will also reduce the interference artifacts produced by laser reflection on the surface (Rico et al. 2013; Yu et al. 2017). Indeed, given the micrometer dimensions of HS-FS cantilevers, part of the laser light reaches the sample surface leading to interference patterns, especially on reflective surfaces such as gold. To reduce interferences, the optical laser can be substituted by a superluminescent diode (SLED) (Rico et al. 2013). While gold substrate were broadly used to easily anchored molecules using thiol-based chemistry, they are nowadays be substituted by non-reflective surfaces such as glass or mica using PEG linkers and site-directed immobilization strategies (Sumbul et al. 2018a; Schoeler et al. 2014; Yu et al. 2017; Yin et al. 2005; Otten et al. 2014; Durner et al. 2017; Sumbul et al. 2018b). Nevertheless, interference artifacts, although minimized, are still present on glass or mica surfaces. Thus, reduction of optical interference remains an important pending improvement for accurate force control. Finally, the force resolution of ultrashort cantilevers is still far from that of optical tweezers, which already achieve time resolution of ~ 10 μs at sub-pN force levels (Neupane et al. 2016), and is a limiting factor when probing the mechanics of labile systems. As described above, milled cantilevers offer improved force precision, but further reduction of the viscous drag coefficient seems to be still necessary. As we attain the diffraction limit of the laser spot size, this may require a radically new approach of force sensing.

## Force measurements at microsecond timescales on single molecules and cells

HS-FS or microsecond force measurements have provided important insights on single molecules mechanics, particularly in protein unfolding. It has enabled an accurate description of the protein-unfolding and bond-unbinding pathways on old and well-studied protein systems by revealing closely spaced and/or transiently occupied intermediate states previously undetected (Florin et al. 1994; Merkel et al. 1999; Oesterhelt et al. 2000).

**Fig. 2:**
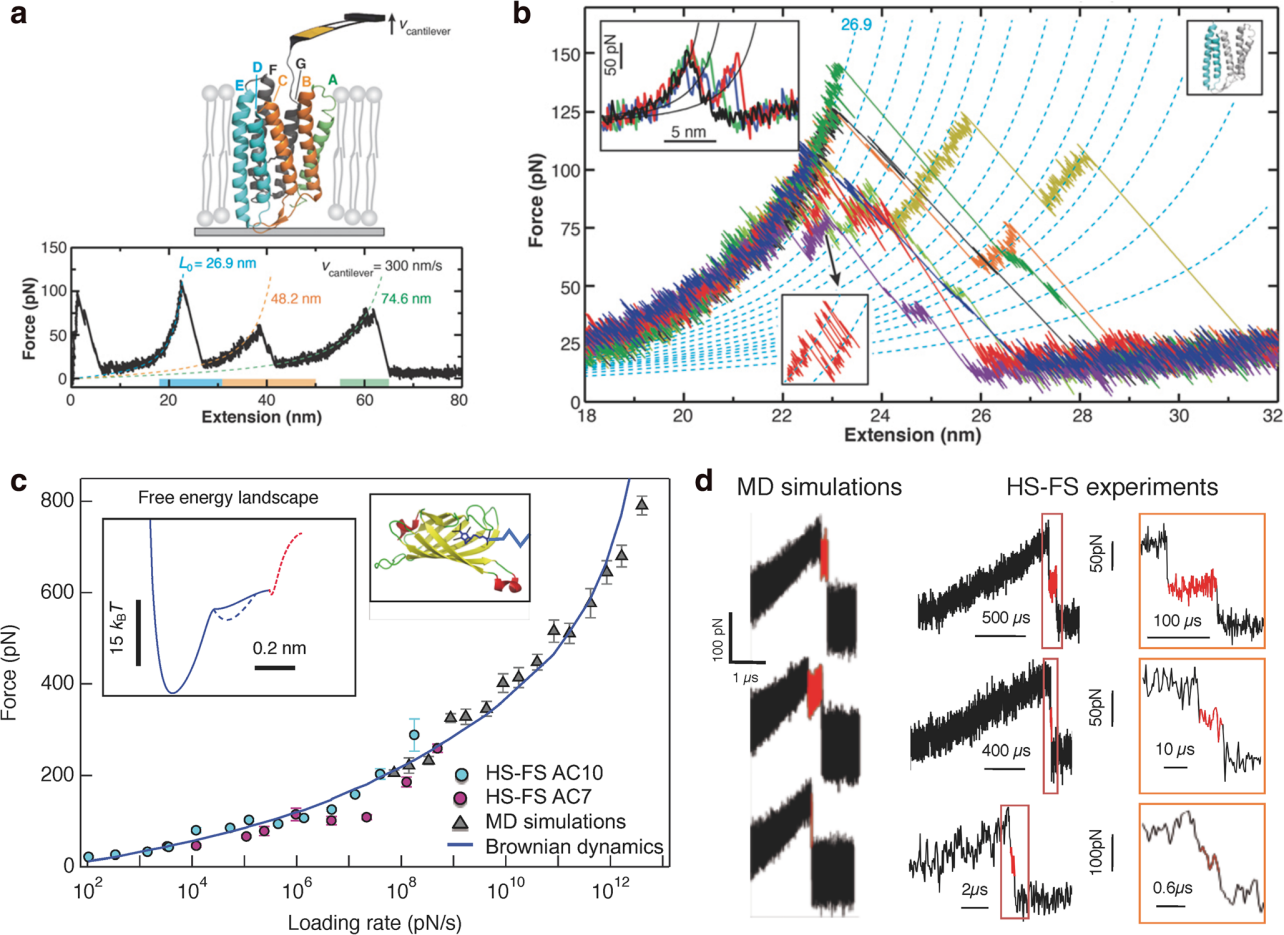
High-speed force spectroscopy of single molecules. **a** Unfolding of bacteriorhodopsin using conventional AFM cantilevers revealed three main unfolding peaks, corresponding to helices A, CB and ED. **b** Force-extension curves of bacteriorhodopsin at 1 μs time resolution reveal 14 intermediates when unfolding the ED helix (top right inset), including refolding events (bottom inset), while only two intermediates were reported in prior studies (top left inset) (reprinted from Yu et al. 2017) **c** Dynamic force spectrum of streptavidin-biotin unbinding. Rupture forces (± SEM) from HS-FS experiments (circles) and MD simulations (triangles). The blue line represents a Brownian dynamics fit to the whole force spectrum describing a free energy landscape with two main barriers (blue line, insert plot). **d** MD and HS-FS force curves with sub-μs time resolution revealed short binding states out of the binding pocket signature of outer barriers (red line in C, insert plot) (reprinted from Rico et al. 2019)

Bacteriorhodopsin (BR), a model membrane protein formed by seven transmembrane helices and homologous to biomedically important G protein-coupled receptors (GPCRs, important drug targets), has been extensively studied (Oesterhelt et al. 2000). Yu et al. (2017) applied HS-FS to this canonical system with the aim of quantifying the energetics of individual membrane proteins embedded in a native lipid bilayer. Force extension curves recorded at 300 nm/s (Fig. 2a) revealed the three major intermediates, corresponding to the unfolding of helices ED, CB, and A, described in the consensus unfolding pathway emerged over the past 15 years (Oesterhelt et al. 2000). However closer inspection of the unfolding traces uncovered “hidden” intermediates and a complex dynamic (un)folding network (Fig. 2b). For instance, while only two nonobligate intermediates were described during unfolding the ED helix pair over the past 15 years (Fig. 2b; left inset), HS-FS enabled to observe 14 intermediates. Changes in contour length between states could be derived from WLC fits to the data, with an estimated accuracy along the polypeptide of ± 1 amino acid. Transitions corresponding to the unwinding of just two amino acids and dwell times as short as 8 ms could be resolved. The higher resolution of HS-FS (~ 100 fold improvement in temporal resolution and ~ 10 fold improvement in force precision in comparison with previous studies on BR) allowed detection of rapid, reversible back-and-forth transitions between two or even three states (Fig. 2b, bottom inset) while stretching. This indicates that the structural elements associated with BR unfolding are smaller than assumed from previous experiments (two or three amino acids *vs*. pairs of helices, single helix, or approximately half a helix) but these small changes in the molecular conformation are too fast to be detected in previous SMFS experiments due to the limited force and time resolution. The wide spreading of refolding events also suggests that the mechanical unfolding of BR at standard stretching rates is likely to occur close to equilibrium but usually masked by experimental limitations when using standard force probes.

As reported by Takahashi et al., refolding signatures were also observed while unfolding spectrin, a three α-helix bundle present in the cell cytoskeleton (Takahashi et al. 2018). Combination of HS-FS up to ~ 15 mm/s and MD simulations down to 5 mm/s revealed the viscoelastic molecular force buffering function of this protein due to a combined mechanism of bundle unfolding and α-helix unwinding. Indeed, while at low velocities (≤ 100 μm/s) most traces exhibited a rather flat force plateau of ∼ 25 pN, at high velocity (≥ 100 μm/s) they displayed more often individual force peaks corresponding to collapse of the tertiary structure of spectrin repeats. The unfolding peaks were well described by an irreversible barrier crossing model while the force plateau spectrum agreed with a near-equilibrium model where unfolding and refolding are possible, disruption and reformation of α-helical hydrogen bonds competing over the stretching pathway. MD simulations supported this interpretation revealing unwinding-rewinding processes even at 5 mm/s stretching velocities. Thus, HS-FS combined with molecular simulations revealed that spectrin is acting as a soft spring at short extensions (70–100 Å) while it is showing a viscous response at larger extensions (100–300 Å, *i.e*., about 5 times the folded length of spectrin).

HS-FS was also combined with MD simulations to provide detailed and dynamic description of receptor-ligand bonds and their (un)binding pathways (Rico et al. 2019). For instance, exploration of the prototypical streptavidin–biotin complex over a large dynamic range of loading rates (11 decades, from 10^2^ to 10^13^ pN/s, achieving tip velocities up to 30 mm/s, more than two orders of magnitude faster than conventional AFM) enabled accurate reconstruction of the unbinding free-energy landscape (Fig. 2c, left inset). The excellent agreement between experiments and fully atomistic explicit solvent simulations at overlapping velocities (Fig. 2c) provided the most direct test of the “computational microscope” and evidenced the unbinding mechanisms at atomistic level. Moreover, transient events far from the binding pocket revealed by MD simulations were supported by the observation of sub-μs binding events using HS-FS. The results stressed out the need of developing theoretical models to consider the dynamic nature of biological bonds. While protein–ligand interactions were commonly described as a unique unbinding pathway, combined HS-FS and MD revealed that biotin-streptavidin unbinding is governed by multiple pathways modulated by transient, timescale-dependent induced fits (Fig. 2d). Indeed, during unbinding, biotin crosses multiple energy barriers while streptavidin binding pocket undergoes nonequilibrium conformational changes that depend on the loading rate. This multi-step unbinding process, where several intermediate conformations have to be visited before the final release, slows down the unbinding of the two molecules and therefore enhances rebinding, thus explaining the long lifetime of the complex.

To quantify mechanical properties of cells, HS-FS had to tackle additional challenges. The correction of viscous drag effects is particularly important when probing soft viscoelastic samples such as eukaryotic cells with a Young’s modulus of about 10 kPa or less. Moreover, large scan-range is required to ensure that the tip lifts off the surface in between the force curves. Using short cantilevers, Braunsmann et al. (2014) performed force mapping on living mouse embryonic fibroblasts with a resolution of 128 × 128 pixels in less than 1.5 min per map, so 10 to 100 times faster than conventional force mapping. This allows resolving dynamic processes such as cytoskeleton reorganization during cell locomotion, growth of individual cytoskeleton fibers, cell blebbing, and formation of endocytic pits in the cell membrane, while mapping cell elasticity. The force curve rate was increased from 2 to 300 Hz, which facilitated force mapping measurements at high speed, and resulted in an increase of the measured apparent Young’s modulus by about 10 times.

**Fig. 3:**
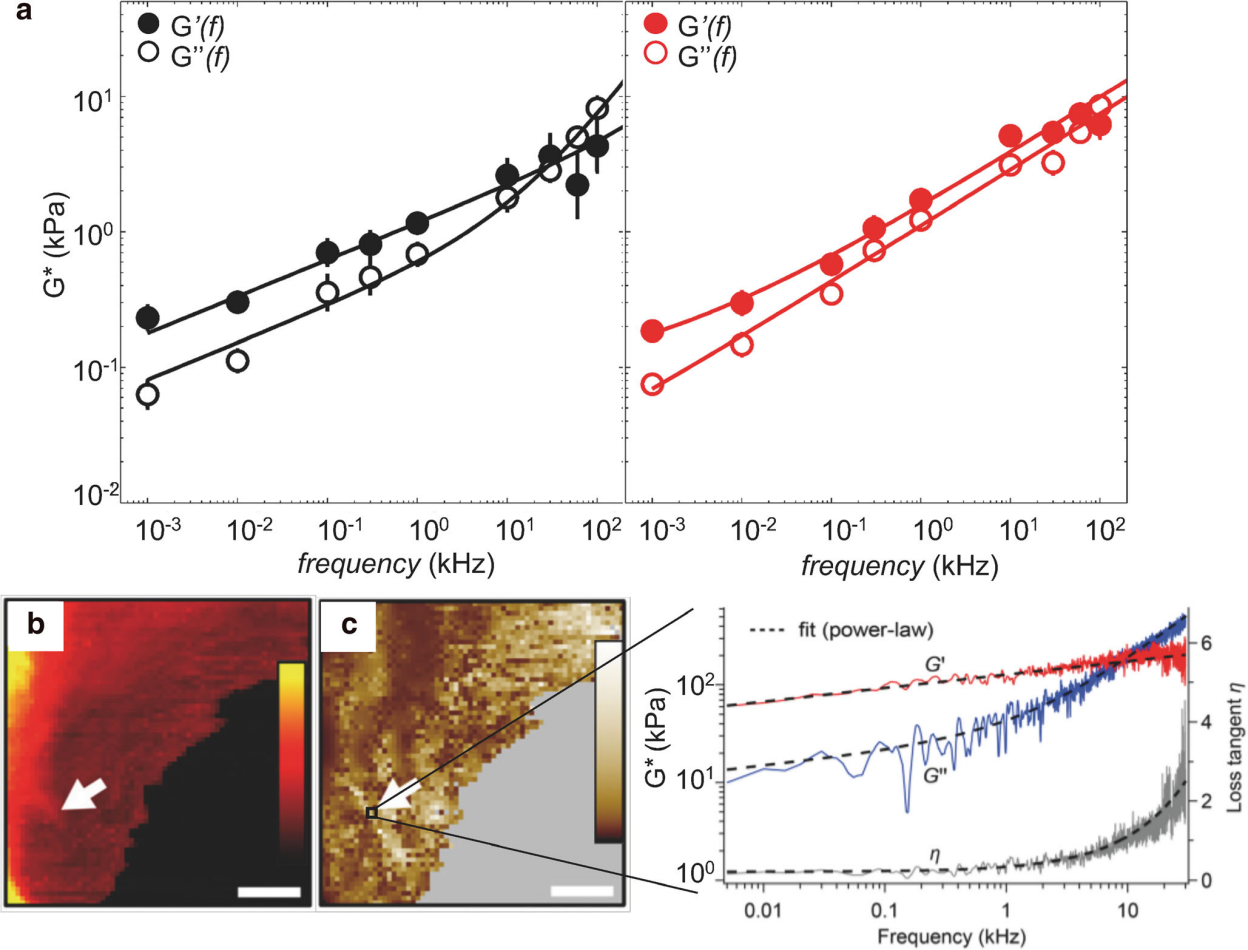
HS-FS enables to explore the mechanical behaviors of cells over a large range of frequency. **a** Frequency-dependent complex shear modulus of benign (black) and malignant (red) cancer cells. The response was well described by two power law regimes, one with weak exponent (0.05–0.2) corresponding to cytoskeleton dynamics at the mesoscale and a stronger exponent (0.4–1) interpreted in terms of semiflexible filament dynamics (reprinted from Rigato et al. 2017). Maps of **b** height and **c** shear modulus (G_0_) on a live fibroblast (scale bars 3 μm, 64 × 64 pixels). The G_0_ map was built by analyzing the frequency dependant of the storage and loss moduli (right plot, G′, red, and G″, blue) and loss tangent (*η*, gray) in a frequency range of 5 Hz–30 kHz at each pixel (inset curve) (reprinted from Schachtele et al. 2018)

Indeed, HS-FS enables to probe living cells at short time scales at which they exhibit different mechanical behavior. By performing microrheology measurements over a large frequency range (low amplitude oscillations from 1 Hz to 100 kHz), Rigato et al. (2017) evidenced that cell viscoelasticity presented two dynamic regimes. At timescales longer than a millisecond (*i.e*., at low frequency), the viscoelastic response followed a weak power law dominated by elastic stresses, often interpreted as cytoskeleton deformations at the mesoscale. In contrast, at previously inaccessible shorter timescales (down to 10 μs, or 100 kHz) the viscoelastic response was better described by semiflexible filaments theories. The possibility of applying semiflexible filament theories allows a mechanistic description of the cytoskeleton state. These findings were corroborated by observations on different cell types under different cytoskeletal conditions using drug treatments. Therefore, the cell mechanical response to high-frequency is rich of information and enables us to characterize the molecular mechanisms, reflecting the morphological and dynamical state of the cytoskeleton. Moreover, malignant cancer cells exhibit a unique mechanical fingerprint at high frequency in comparison with healthy cells (Fig. 3a). Thus, probing cells responses over a wide dynamic range may provide a unique characterization of the mechanical phenotype of living cells and give new insights for a better mechanistic understanding of cell mechanics.

High frequency microrheology of living cells was recently improved. Schächtele et al. (2018) developed a new resonance compensating chirp mode (RCCM) to probe the frequency dependence of the viscoelasticity of living cells (quasi-) continuously over a large frequency range (5 Hz–30 kHz) and with a resolution of 5 Hz. A continuous frequency sweep (chirp) is applied to the z-scanner and is iteratively shaped to compensate for scanner resonances. This allows extending the measurement range far beyond the resonant frequency of the scanner (five times higher). The complex shear modulus of mouse embryonic fibroblast cells exhibited a weak power-law behavior over almost all the frequency range with a stronger frequency dependence at the highest rates (Fig. 3c). A short chirp duration (200 ms) and a high approach velocity (320 m/s) allowed mapping live cells and generating spatially resolved images of the power-law parameters (Fig. 3b) within minutes (≈ 20 min for 64 × 64 pixels), revealing local viscoelastic variations.

## Conclusions and future perspectives

Ultrashort cantilevers and HS-AFM set-ups allow force measurement with sub-microsecond time resolution, providing important insights on both cellular and molecular mechanics. By reaching pulling velocities about two orders of magnitude higher than conventional AFM, HS-FS bridges the gap between experimental force measurements and MD simulations, enabling an atomic description of molecular processes supported by experimental data. The improved time resolution allows observation of hidden intermediate states that reveal the complex energy landscapes of biomolecular processes. Thus, HS-FS provides a framework to better understand theory, experiments, and simulations. While many applications have revealed the complexity of the mechanics of biomolecules and living cells, there are still several exciting challenges to come. This may require further improvements and likely radical changes in AFM technology.

The next plausible step seems to be the application of single molecule HS-FS on living cells to probe receptor-ligand unbinding and/or adhesion molecules unfolding in situ, in the actual physiological environment. The combination of HS-AFM imaging with mechanical intervention of biomolecules starts also to emerge as a promising interactive approach and its application to living cells may arrive soon (Chiaruttini et al. 2015; Ganser and Uchihashi 2019; Owa et al. 2019). Moreover, current efforts point towards the development of even faster force mapping using ultrashort cantilevers. The groups of Schaeffer and Fantner have carried out significant progress in this direction by developing fast force mapping on cells and photothermal off-resonance using ultrashort levers (Schaeffer et al. 1997; Nievergelt et al. 2018). We expect that the use of ultrashort cantilevers as a force probe with sub-μs response time will grow and continue revealing fast processes in biomolecules and cells.
